# Monthly Summary

**Published:** 1887-03

**Authors:** 


### Monthly Summary.

---

CHLORAL HYDRATE AS A VESICANT.—Attention has quite recently been called to the vesicant properties of chloral hydrate. According to the *Medical Press*, for blistering purposes, chloral hydrate is fully as efficacious as cantharides, while it is free from the inconveniences attending the employment of this latter agent. The chloral should be reduced to a powder, and a layer of it placed on a piece of common adhesive plaster, taking care to leave a margin between the edge of the layer of chloral and that of the plaster. This is then warmed over a gas jet until the chloral becomes discolored and melts, when it should be immediately applied on the spot for the operation, the skin covering which is to be anointed beforehand with olive oil or lard. The anæsthetic properties of the chloral prevent any unpleasant sensation, and fifteen minutes is the maximum period of time during which the application may be continued. If the above mentioned precaution be taken of anointing the skin, its vitality is retained, and the presence of an open wound is avoided, the skin adhering again as soon as the exudation is evacuated. Another advantage consists in the absence of the risk of poisonous effects, consequent on absorption, a by no means uncommon sequel to the use of cantharides.

---

A DEAR KISS.—A short time ago, a Berlin dentist was brought before a magistrate for kissing a young lady after a dental operation. The court considered this action such a grave breach of confidence that they punished him with three months' imprisonment. In America or England, a young lady under such circumstances would have received $1,000; but, should such a course be pursued in Germany, no dentist would be safe from the visits of ladies anxious to obtain dowries at so slight a cost. Any way, the sentence was not undeserved, for it is of the first importance in our profession that the confidence of the public should remain unshaken.

Some years ago the laws of morality suffered a greater outrage at the hands of a Berlin dentist; and these cases may serve as warning beacons to those engaged in the profession of dentistry.—*Die Zahntechnische Reform.*

---

INTERNATIONAL MEDICAL CONGRESS.—Prof. J. J. Chisolm of the University of Maryland, has been appointed President of the Section of Opthalmology in place of Dr. E. Williams, who resigned on account of ill-health. Dr. Judson B. Andrews, Superintendent of the Buffalo, N. Y,, Insane Hospital, has been appointed President of the Section of Psychological Medicine and Nervous Diseases, made vacant by the death of Dr. John P. Gray. There are now no vacancies in the list of chief officers of the preliminary organization of the congress or its sections.

